# Complete Genome Sequence of *Sulfurospirillum* Strain ACS_TCE_, a Tetrachloroethene-Respiring Anaerobe Isolated from Contaminated Soil

**DOI:** 10.1128/MRA.00941-20

**Published:** 2020-10-01

**Authors:** Leitao Huo, Yi Yang, Yan Lv, Xiuying Li, Frank E. Löffler, Jun Yan

**Affiliations:** a Key Laboratory of Pollution Ecology and Environmental Engineering, Institute of Applied Ecology, Chinese Academy of Sciences, Shenyang, Liaoning, China; b University of Chinese Academy of Sciences, Beijing, China; c Department of Microbiology, University of Tennessee, Knoxville, Tennessee, USA; d Center for Environmental Biotechnology, University of Tennessee, Knoxville, Tennessee, USA; e Biosciences Division, Oak Ridge National Laboratory, Oak Ridge, Tennessee, USA; f Joint Institute for Biological Sciences (JIBS), Oak Ridge National Laboratory, Oak Ridge, Tennessee, USA; g Department of Civil and Environmental Engineering, University of Tennessee, Knoxville, Tennessee, USA; h Department of Biosystems Engineering & Soil Science, University of Tennessee, Knoxville, Tennessee, USA; University of Southern California

## Abstract

Here, we report the complete genome sequence of the tetrachloroethene-to-trichloroethene dechlorinator *Sulfurospirillum* sp. strain ACS_TCE_. The genome consists of a 38.05-kb circular plasmid and a 2.69-Mb circular chromosome, which encodes 3 identical reductive dehalogenases with 91.47% amino acid identity to the PceA of Sulfurospirillum multivorans strain DSM 12446.

## ANNOUNCEMENT

Some members of the genus *Sulfurospirillum* have versatile energy metabolisms, including organohalide respiration, a vital process for the turnover of chlorinated compounds in environmental systems (1–4). *Sulfurospirillum* sp. strain ACS_TCE_, which reductively dechlorinates tetrachloroethene (PCE) to trichloroethene, was isolated from a PCE-dechlorinating consortium derived from contaminated soil collected at the Axton Cross Superfund site near Holliston, MA (5). Sanger sequencing revealed that strain ACS_TCE_ shares 98.6% 16S rRNA gene sequence identity with the PCE-to-*cis*-1,2-dichloroethene dechlorinator Sulfurospirillum multivorans strain DSM 12446 (4, 5).

Strain ACS_TCE_ was grown in anoxic, bicarbonate-buffered mineral salt medium under PCE-dechlorinating conditions (5). Cells were harvested by centrifugation at 21,000 × *g* for 30 min, and genomic DNA was extracted using the cetyltrimethylammonium bromide method (6). The genome was sequenced using a dual-platform approach. For Illumina sequencing, DNA was sonicated to generate <500-bp fragments, followed by T-A ligation to add adaptors and library preparation using the VAHTS universal DNA library prep kit (Vazyme Biotech Co., Nanjing, China) following the manufacturer’s instructions. Paired-end sequencing (2 × 150 bp) of the DNA library with an average insert size of 350 bp was performed on a HiSeq 2000 instrument (Illumina, San Diego, CA, USA). For PacBio sequencing, DNA was sheared using g-TUBEs (Covaris, Inc., Woburn, WA, USA) to generate 10-kb fragments, which were subsequently end repaired and ligated with universal hairpin adapters using the SMRTbell template prep kit 1.0 (Pacific Biosciences, Menlo Park, CA, USA) following the manufacturer’s instructions. The library was sequenced in a single single-molecule real-time (SMRT) cell using a PacBio RS II/Sequel SMRT instrument (7). The PacBio raw read *N*_50_ value is 2,794 bp. The genome sequence was assembled with 840,203 PacBio raw long reads (coverage, 712×) using WGS-Assembler version 8.2 and polished with 12,672,060 Illumina short reads (coverage, 701×) using Pilon version 1.22 (8, 9). Circlator version 1.5.1 was used to evaluate genome completeness, remove overlapping ends, and circulate the genome (10). Default parameters were used for all software unless otherwise specified. Functional annotation was performed using the NCBI Prokaryotic Genome Annotation Pipeline (PGAP) (11).

The final assembly resulted in a 2,685,870-bp circular chromosome and a 38,046-bp circular plasmid (Table 1). Using the genome of *S. multivorans* strain DSM 12446 (GenBank accession number CP007201.1) as a reference, the replication origin in the chromosome of strain ACS_TCE_ was rotated to the 159-bp position (noncoding region) upstream of the *dnaA* gene, which encodes the replication initiator protein DnaA. The strain ACS_TCE_ chromosome harbors two rRNA operons organized in the order 16S-23S-5S. Four reductive dehalogenase (RDase) A genes, of which three are identical and adjacent to a downstream RDase B gene encoding a membrane-bound anchor protein, are present on the chromosome. The protein encoded by the three identical RDase A genes shares 91.47% amino acid identity with the PceA RDase (GenBank accession number AHJ12791) of *S. multivorans* strain DSM 12446 (12, 13). The chromosome also harbors complete *nap* and *nrf* clusters and a fumarate reductase gene (*frdA*), suggesting other electron acceptors support growth. On the strain ACS_TCE_ plasmid, genes encoding the plasmid replication initiator protein RepB (FA592_13925) and subunits (FA592_13805 and FA592_13810) of a type II toxin-antitoxin system were functionally annotated by PGAP. The majority of plasmid-associated genes encode proteins with unknown function. The genome and novel RDase genes reported in this study can be used to better understand the physiology and evolution in organohalide-respiring *Sulfurospirillum* members.

**TABLE 1:** Genome features of strain ACS_TCE_

Feature	Data for:	
	Chromosome	Plasmid
Assembly length (bp)	2,685,870	38,046
G+C content (%)	38.84	35.71
No. of assembled contigs	1	1
No. of coding sequences	2,704	62
No. of tRNAs	45	0
No. of rRNAs (5S, 16S, 23S)	6 (2, 2, 2)	0
GenBank accession no.	CP045453	CP045454
BioSample accession no.	SAMN11478962	SAMN11478962
BioProject accession no.	PRJNA534163	PRJNA534163

### Data availability.

The genome sequences and assembly projects reported in this article have been deposited in DDBJ/ENA/GenBank under the accession numbers listed in Table 1. Raw reads have been deposited in the Sequence Read Archive (SRA) under accession numbers SRR12303098 (Illumina) and SRR12303097 (PacBio).
